# The Antimicrobial Effects of Saudi Sumra Honey against Drug Resistant Pathogens: Phytochemical Analysis, Antibiofilm, Anti-Quorum Sensing, and Antioxidant Activities

**DOI:** 10.3390/ph15101212

**Published:** 2022-09-30

**Authors:** Abdulrahman S. Bazaid, Abdu Aldarhami, Mitesh Patel, Mohd Adnan, Assia Hamdi, Mejdi Snoussi, Husam Qanash, Mohammed Imam, Mohammad Khalil Monjed, Aiah Mustafa Khateb

**Affiliations:** 1 Department of Medical Laboratory Science, College of Applied Medical Sciences, University of Ha’il, Hail 55476, Saudi Arabia; 2 Department of Medical Microbiology, Qunfudah Faculty of Medicine, Umm Al-Qura University, Al-Qunfudah 21961, Saudi Arabia; 3 Department of Biotechnology, Parul Institute of Applied Sciences and Centre of Research for Development, Parul University, Vadodara 391760, India; 4 Department of Biology, College of Science, University of Ha’il, Hail 81451, Saudi Arabia; 5 Laboratory of Chemical, Pharmaceutical and Pharmacological Development of Drugs, Faculty of Pharmacy, Monastir 5000, Tunisia; 6 Laboratory of Genetics, Biodiversity and Valorization of Bio-Resources, Higher Institute of Biotechnology of Monastir, University of Monastir, Avenue Tahar Haddad, BP74, Monastir 5000, Tunisia; 7 Molecular Diagnostics and Personalized Therapeutics Unit, University of Ha’il, Hail 55476, Saudi Arabia; 8 Department of Biology, Faculty of Applied Science, Umm Al-Qura University, Makkah 21961, Saudi Arabia; 9 Medical Laboratory Technology Department, College of Applied Medical Science, Taibah University, Madinah 42353, Saudi Arabia; 10 Special Infectious Agents Unit-BSL3, King Fahd Medical Research Center, King Abdulaziz University, Jeddah 21362, Saudi Arabia

**Keywords:** Sumra honey, antibacterial, antifungal, antioxidants, antibiofilm, anti-quorum-sensing

## Abstract

Honey exhibited potential antimicrobial activity against multidrug resistant (MDR) bacteria that continues to be a serious health problem. We reported the in-vitro activity of Saudi Sumra honey against clinical pathogenic bacteria and fungi, antibiofilm, anti-quorum-sensing (QS) and antioxidant activities in relation to its phytochemical composition assessed by gas chromatography-mass spectrometry (GC-MS). Broth dilution method and scavenging activities against 2,2-diphenyl-1-picryl-hydrazyl-hydrate (DPPH) and 2,2′-azino-bis (3-ethylbenzothiazoline-6-sulfonic acid) (ABTS) and *β*-carotene bleaching assays were performed. The GC-MS analysis of Sumra honey showed that 2,4-dihydroxy-2,5-dimethyl-3(2H)-furan-3-one 1-methylcyclopropanemethanol were the major identified phytoconstituents. Sumra honey showed a minimum inhibitory concentration (MIC) to clinical isolates of *Staphylococcus aureus* including methicillin-resistant *Staphylococcus aureus* (MRSA) at 300 mg/mL, *Pseudomonas aeruginosa* (250 mg/mL), *Escherichia coli* (350 mg/mL) and *Acinetobacter baumannii* (250 mg/mL); clinical fungal isolates—*Candida auris* (600 mg/mL) and *Cryptococcus neoformans* (>1000 mg/mL); wild type fungal isolates—*Candida krusei* (>1000 mg/mL) and *Candida albicans* (700 mg/mL). In addition, Sumra honey demonstrated promising inhibition targeting biofilm formation by 59% for *Bacillus subtilis*, 48% for *S. aureus*, 38% for *E. coli*, and 33.63% for *P. aeruginosa*. The violacein production in *Chromobacterium violaceum* was reduced to 68%, whereas pyocyanin production in *P. aeruginosa* was reduced to 54.86% at ½ MIC. Furthermore, Sumra honey exhibited strong antioxidant activities (DPPH − IC_50_ = 7.7 mg/mL; ABTS − IC_50_ = 5.4 mg/mL; *β*-carotene − IC_50_ = >20 mg/mL). Overall, obtained data highlighted the promising potential therapeutic use of Sumra honey treating infections caused by MDR bacteria and fungi. Moreover, Sumra honey can be a good candidate as an inhibitor agent for bacterial cellular communication in strains of *P. aeruginosa* and *C. violaceum*.

## 1. Introduction

It is widely reported that antimicrobial resistance (AMR) is posing a very serious threat to public health worldwide [1]. This is because multi-drug resistant (MDR) bacteria carries the highest rate of morbidity, serious complications, and mortality since affective drugs are not available or very limited [1]. AMR has been on the rise worldwide for the past several years, including the last-resort drugs that are regularly prescribed [2]. Globally, the number of deaths associated with AMR is more than 700,000 annually, which is expected to reach 10 million by 2050, unless targeted actions are taken before then [1]. It was estimated that approximately 2.8 million cases and 35,000 deaths per year are resulted from AMR in the United States only [3]. In addition, there are more than 670,000 annual cases and 33,000 deaths that are associated with AMR in Europe [4]. In Saudi Arabia, incidences of AMR are increasing, commonly towards *Klebsiella* *pneumoniae*, *Escherichia coli*, *Pseudomonas aeruginosa*, and *Staphylococcus aureus* [5,6,7]. Therefore, urgent actions are required to tackle the potential crisis of antibiotic resistance nationally and globally. These actions may include public awareness, developing new antibiotics, control of the use of antibiotics (antibiotic stewardship), and use of antibiotic alternative approaches [8].

Antibiotic alternative approaches would include bacteriophages (viruses that lyse bacteria) [9], antimicrobial peptides (AMPs) [10], or natural products, such as plant extract, essential oils, and honey [11,12,13]. Different types of natural honey have been reported for their antimicrobial activity against a broad range of bacteria and, thus, they can be used to treat bacterial infections caused by MDR bacteria. The exact mechanism of natural honey’s antimicrobial action is still unclear/inconclusive, but it is widely believed that there are multiple underlying mechanisms attributed to its antimicrobial activity [11]. Nevertheless, honey is expected to eradicate bacteria by one or more of the following modes of action: disrupting/damaging bacterial cell membrane, inhibiting bacterial virulence factors, or preventing bacterial adhesion to host cells [14,15]. An additional feature of honey, unlike other antimicrobial agents, is that microbial resistance to honey is not widely reported in the literature as it has not been well established yet [16].

The Saudi Acacia honey, also known as Sumra honey, is one of the darkest, thickest, and richest honeys in the world. It is dark brown with amber undertones that has a flavor profile of smokey nutty sweetness [17]. It is collected from the nectar of *Acacia* trees, which are only found in the desert areas of Arabia. It is considered a rare honey, as it is only harvested twice yearly. In particular, Sumra honey is very popular in Arab culture, which might be due to its potential antioxidant, anti-inflammatory, and anti-bacterial properties [17]. Providing more information about the antimicrobial activity of such honey, especially against MDR pathogens, might be essential for the evaluation of its potential application in clinical settings. Therefore, the focus of the present study was to conduct phytochemical analysis and to further examine in depth the antimicrobial, antioxidant, anti-quorum sensing, and antibiofilm activities of Sumra honey.

## 2. Results

### 2.1. Antibacterial Potential of Sumra Honey

The potential antagonistic activity of Sumra honey was quantitatively studied against different clinical bacterial isolates (Methicillin-resistant *Staphylococcus aureus* [MRSA (1) and MRSA (2)], *P. aeruginosa*, *E. coli* and *A. baumannii*) and reference bacterial strains (*B. subtilis*, *S. aureus*, *E. coli* and *P. aeruginosa*). Upon testing for the antibacterial activity, Sumra honey showed a broad-spectrum antibacterial activity against tested Gram-positive and negative bacteria. It is widely recognized that MIC and MBC evaluation is a good and comparatively economical way to simultaneously assess multiple antimicrobial agents for their effectiveness. The MIC and MBC values of Sumra honey against clinical isolates were higher than those of their reference counterpart, 250 and 300 mg/mL for *P. aeruginosa*, 250 and 450 mg/mL for *A. baumannii*, 250 and >450 mg/mL for MRSA-2, 300 and 350 for MSSA, 300 and >450 for MRSA-1, 350 and >450 for *E. coli*, whereas the MIC and MBC values of Sumra honey towards reference bacterial strains were as follows: 80 and 100 mg/mL for *B. subtilis*, 90 and 150 mg/mL for *S. aureus*, 100 and 200 mg/mL for *E. coli*, and 120 and 200 mg/mL for *P. aeruginosa*, respectively (Table 1). The MBC/MIC ratio of Sumra honey against all tested bacteria were always approximately two-fold higher than their MIC values, suggesting that Sumra honey possesses a bactericidal effect against all tested bacteria.

### 2.2. Antifungal Activity of Sumra Honey

Sumra honey showed increasing inhibitory activity with increasing honey concentrations against some tested fungal strains. Among them *Candida auris* and *Candida albicans* had the lowest MIC level at 600 and 700 mg/mL of Sumra honey, respectively. However, *Cryptococcus* *neoformans* and *Candida krusei* grew at the highest honey concentration (1000 mg/mL) and showed turbidity similar to a positive control (Table 2).

### 2.3. Antibiofilm Properties of Sumra Honey

**Figure 1:**
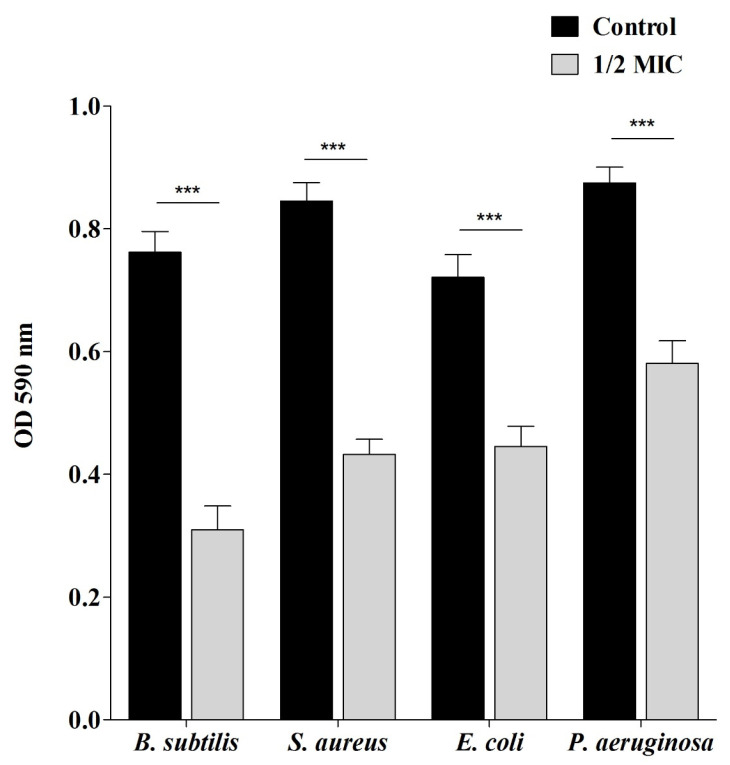
Antibiofilm activity of Sumra honey against four bacterial strains. Sumra honey inhibited the biofilm formation of treated bacteria in comparison with the control, evidenced by reduced optical density. Error bars indicate SDs (±standard deviation) of three independent experiments; Significance; *** *p* < 0.0001.

To determine the antibiofilm ability of Sumra honey, it was assessed against reference bacterial strains via its ability to affect their adhesion to the surface. Data showed that Sumra honey was able to inhibit the adherence of tested bacteria to the surfaces at the ½ MIC. At this concentration, the adhesion ability was decreased with the percentage of inhibition as 59.38% for *B. subtilis*, 48.83% for *S. aureus*, 38.25% for *E. coli*, and 33.63% for *P. aeruginosa* (Figure 1).

### 2.4. Anti-Quorum Sensing Properties of Sumra Honey

**Figure 2:**
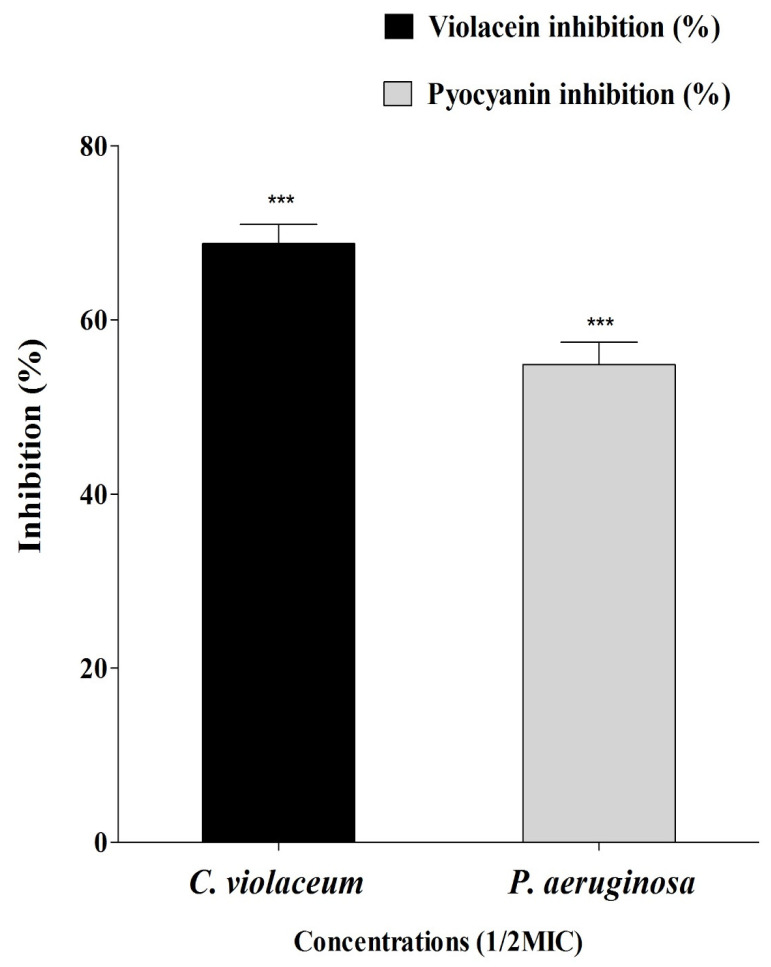
Anti-quorum sensing properties of Sumra honey against two bacterial strains. Error bars indicate SDs (±standard deviation) of three independent experiments. Bars with *** indicates statistically significant (*p* < 0.0001) compared to control.

Sumra honey was assessed for quorum sensing modulatory properties against *Chromobacterium violaceum* and *P. aeruginosa*. After treating both tested bacteria with the Sumra honey, a significant reduction in pigment production was observed in both bacteria, which is indicative of a reduction in their growth as well as the presence of anti-QS activity due to its strong inhibitory effect. The MIC values of Sumra honey against *C. violaceum* and *P. aeruginosa* were 80 mg/mL and 120 mg/mL, respectively. As a result of the Sumra honey interfering with the enzyme of QS activity, the violacein and pyocyanin pigment production in *C. violaceum* and *P. aeruginosa* decreased. Violacein production in *C. violaceum* was reduced to 68.73%, whereas pyocyanin production in *P. aeruginosa* was reduced to 54.86% at ½ MIC of Sumra honey (Figure 2).

### 2.5. Antioxidant Properties of Sumra Honey

Due to the level of diversity of bioactive compounds present in the tested honey, DPPH, ABTS and *β*-carotene bleaching methods were undertaken to evaluate the antioxidant capacity of Sumra honey. Sumra honey and a commercialized standard (ascorbic acid) was screened for its ability to scavenge free radicals (Table 3). The half-maximal inhibitory concentration IC_50_ values of Sumra honey and the standard, which indicate the concentrations required for scavenging half (50%) or more of the tested radicals, demonstrated that DPPH and ABTS were strongly inhibited by Sumra honey. The IC50 values of Sumra honey towards DPPH and ABTS were monitored as 7.7 mg/mL and 5.4 mg/mL, respectively.

### 2.6. Identification of Bioactive Constituents from Sumra Honey by Gas Chromatography-Mass Spectrometry (GC-MS)

Sumra honey was analyzed using GC-MS methods to determine the present bioactive constituents. Chemical components identified in Sumra honey are listed in Table 4 (Supplement Figure S1). Based on the total ion chromatographic analysis of Sumra honey, it was observed that Sumra honey contains a variety of potentially active components with varying retention times. By using mass spectrometry (MS) analysis, it was possible to recognize the identified structural components. The main identified bioactive metabolites were 5-methyl-2-ethylamino-2-thiazoline, 4-Hydroxy-1-[4-(hydroxymethyl)-3,6- dioxabicy-clo [3.1.0]hexan-2-yl]-5-methylpyrimidin-2-one, 2-chloro-Propanoic acid and Cyclohexanone (Table 4).

## 3. Discussion

There is a growing demand to find alternative agents/approaches to replace antibiotics due to the global threat of AMR, which continues to increase despite the limited number of effective antibiotics targeting MDR bacteria [1]. Consequently, a high demand towards various natural products (NPs) presented with antibacterial activity and mode of action unlike conventional drugs, including honey, was widely observed [11,18]. Honey has been revered and valued as one of nature’s greatest gifts. In traditional medicine, honey is used for wound healing as well as treating cancer and other clinical conditions as an alternative to conventional treatment. There are many populations around the world that have used honey for both medicinal and nutritional purposes. Honey is used to treat a wide range of diseases conditions, including eye diseases, bronchial asthma, tuberculosis, hepatitis, throat infections, piles, eczema, wounds, prompt ulcers, and wound healing, in addition to its dietary benefit [19]. Honey has been found to possess antioxidant, antimicrobial, anti-inflammatory, antiproliferative, anticancer, and antimetastatic effects [19]. Several studies have shown that honey can be used to treat wounds, diabetes mellitus, cancer, and cardiovascular, neurological, and gastrointestinal diseases. The antibacterial properties of honey have been studied over the years, and many studies have found that natural honey has some broad-spectrum antibacterial activity towards drug resistant pathogenic bacteria [11]. Therefore, honey has been considered as a potential bioactive natural product that showed promising activity against pathogenic bacteria to treat a variety of bacterial infections [20]. Due to the fact that different types of honey possess a wide range of antibacterial activity, such activity appears to be independent of antibiotic susceptibility or resistance and appears unlikely to turn pathogenic bacteria resistant to honey [21,22]. Different types of Saudi honey showed a significant inhibitory effect towards MRSA and MSSA strains [11]. However, studies focusing on the bioactive properties of Sumra honey are limited compared to other honeys, and thus the current study aimed to fill this data gap by evaluating phytochemical analysis and the antibacterial, antioxidant, and antibiofilm activities of Sumra honey.

Obtained data indicated that Sumra honey exhibits broad spectrum antibacterial activity against all tested isolates of both Gram-positive and negative bacteria. The antibacterial activity of Sumra honey was found to be higher against reference strains (MTCC) in comparison to clinical isolates. This can be attributed to the fact that clinical isolates are exposed to various antimicrobials with optimal, sub-lethal, and overdoses in comparison with reference strains, thus more selection pressure for resistance would be highly expected. Many pathogenic micro-organisms, including *Enterobacter aerogenes*, *S. aureus*, and *Salmonella typhimurium*, have been inhibited by Manuka honey [23]. Honey has been found to be effective against drug resistant isolates including MRSA, drug resistant hemolytic *Streptococci*, and vancomycin-resistant *Enterococci* (VRE) [24]. In addition, a variety of honey from different regions of the world may have similar or better potency than Manuka honey. There are few studies that have investigated the bioactive potential of traditional Saudi honeys. Using different Saudi honey samples, Hegazi and colleges found that they were effective antibacterials against different pathogenic bacteria [25]. Another study demonstrated the antimicrobial activity of 10 honey samples collected from various floral areas surrounding Riyadh [26]. Halawani and Shohayeb examined the effect of the nine honeys commonly used in Saudi Arabia (Sidr honey, Taify Sidr honey, Kashmiri Sidr honey, Shaoka honey, Sumra honey, Black Seed honey, Black Forest honey, and Clover honey) and the effect of Manuka honey against *E. coli*, *P. aeruginosa*, *K. pneumoniae*, *Salmonella enterica*, *Shigella flexneri*, *P. aeruginosa*, and *Streptococcus pyogenes* are the most sensitive bacterial species among tested Gram-negative and -positive bacteria, respectively [27].

The antimicrobial activity of honey can be attributed to several physicochemical properties, including high sugar content, high viscosity, high osmotic pressure, low pH, low water activity, lack of protein content and the presence of hydrogen peroxide [28]. Hydrogen peroxide, which is produced by glucose oxidase action, is the main antibacterial factor found in honey [29]. In the hypopharyngeal glands of the bees, honey-glucosidase is secreted, which breaks glucose molecules to form gluconic acid and hydrogen peroxide. When honey is diluted in water, it restores the glucosidase activity that is inactive due to the little amount of water available and the acidic condition [30]. Nevertheless, it has been demonstrated that in some cases, the antibacterial activity of honey is entirely due to non-peroxide components, such as acidity, osmolarity, flavonoids, phenolic compounds, and lysozyme [31]. Non-peroxide antibacterial activity has the advantage that it remains intact even after storage of honey for long periods of time [32], as well as it does not change under climate or light alterations [33]. In addition to the high honey osmolarity, honey’s low pH and its relative antibacterial activity were attributed to the presence of non-peroxide and non-active components within honey [34]. In the present study, bioactive metabolites known to have antimicrobial potential and different classes of bioactive metabolites including fatty acids, lipids, amino sugars, amino alcohols, small peptides were identified from the Sumra honey via GC-MS analysis. The main identified bioactive metabolite was 5-methyl-2-ethylamino-2-thiazoline. Thiazole derivates exhibited good antimicrobial activity against various Gram-positive including *S. aureus* and four Gram-negative bacteria and limited activity against fungi including *Aspergillus niger* [35]. Moreover, various studies have claimed that honey contains bioactive compounds, such as lysozyme, which possesses well-known antibacterial properties, however some honeys do not exhibit lysozyme activity [36]. The study of Ilyasov et al. has shown for the first time the existence of antibacterial properties in honey whose origin is from a bee, which contains an antimicrobial peptide called defensin [37]. These properties are attributed to the presence of peptides in honey. There has been a recent discovery for the protein Bee defensin-1 in honeybee hemolymph, which has also been found in royal jelly, that provides food for honeybee larvae [38]. In terms of the composition, the physicochemical parameters of honey, such as its acidity and its osmolarity, may be considered as the factors that are the most responsible for its antimicrobial activity [39].

Nevertheless, several chemical compounds present in Sumra honey phytochemicals have already been claimed for its antimicrobial activities. Yaouba et al. have reported the antibacterial activities of alkenyl cyclohexanone against *S. aureus* and *E. coli* [40]. In addition, acetamide was proven for its ability to eradicate the growth of *E. coli*, *Proteus mirabilis*, and *S. pyogenes* [41]. Furthermore, a great sensitivity of *S. aureus*, *E. coli*, and *C. albicans* towards arylhydrazono pyrazoles (AHPs) and aryldiazenyl pyrazoles (ADPs) was observed [42]. Interestingly, 6-Azathymine was reported to inhibit the bacterial growth of *P. aeruginosa* and *E. coli* [43]. Similarly, 2-Furanmethanol was presented with antibacterial and antiviral activities against tested strains of *K. pneumonia* and *S. aureus*, as well as towards bacteriophage MS2 [44]. Other components of Sumra honey were previously tested in-vitro against microbial pathogens with proven antimicrobial activities, including Levoglucosenone, Trispiro [4.2.4.2.4.2.] heneicosane, 2,5-Furandione, Ethanethiol, 2-Amino-2-methyl-1,3-propanediol, and Cirsiumaldehyde [45,46,47,48].

In particular, honey is considered as one of the most potentially effective treatments for biofilm associated infections since previous studies have claimed that honey possesses the potential to both hinder biofilm formation and reduce pre-formed biofilms [49,50]. Current findings show biofilm formation of *Bacillus subtilis*, *S. aureus*, *E. coli*, and *P. aeruginosa* were inhibited by Sumra honey. Overall, Sumra honey revealed a potent antibacterial and antibiofilm effect against a variety of tested bacterial pathogens.

Previous studies have reported a promising broad-spectrum antifungal activity of several honey with great potential to treat fungal infections, particularly opportunistic fungi among immunocompromised patients [51]. At present, Sumra honey demonstrated antifungal activity against tested fungi, including clinical and/or standard laboratory strains. The clinical strain of *C. auris* was the most sensitive isolate among all tested fungi, followed by reference strain of *C. albicans. C. auris* has been associated with multiple epidemics, increasing deaths in hospitals, especially in the intensive care units (ICUs) due to its higher rates of resistance and colonization. Groot et al. showed that *C. auris* was also the most inhibited *Candida* species by medical grade honey formulations compared to *C. albicans* and *Candida glabrata*, demonstrating the antifungal activity of honey [52]. Nevertheless, the level of sensitivity of tested strains of *C. albicans* is inconclusive. *C. albicans* was reported as the least sensitive fungal species to honey samples at several concentrations ranging from 0.1 to 100% [53]. However, Khosravi et al. claimed that different samples of tested honeys were able to produce a complete inhibition for *C. albicans* with MIC ranging from 29 to 56% [54]. Additional reports indicated variable sensitivity of *C. albicans* to different honey samples [55]. In this study, the least sensitive fungal isolates were *C. neoformans* and the reference strain of *C. krusie.* In 2011, Estevinho et al. reported reduced growth rates of *C. albicans*, *C. krusei*, and *C. neoformans* in media contacting monofloral lavender honey samples [55]. *C. krusei* and *C. neoformans* were less sensitive than *C. albicans.* However, level of sensitivity was changed when the used synthetic honey solution, such as *C. albicans* and *C. neoformans*, showed more of a resistance level. Botanical honey origin would play an important role influencing the overall potency of honeys, which could explain these variable values. In addition, emergence of resistant strains, physico-chemical properties, and entomological origin could affect the potency of tested honeys. Future research on honey should be directed towards practical treatment of open wounds or skin that has been colonized by MDR fungal isolates, such as *C. auris*, in a clinical environment.

Quorum sensing (QS) is a process by which the detection and control of gene expression in a population is based on the number of micro-organisms within that population [56]. Pathogenic bacteria use the QS mechanism to develop biofilms. During QS, the signals are exchanged between bacteria via extracellular molecules called autoinducers, which are responsible for sending and receiving signals [56]. The signal molecules are essential for bacteria to express virulence factors, produce secondary metabolites, form biofilms and communicate with other microorganisms [56]. Signal molecules from a bacterium bind to the receptors of other bacterium as part of the QS process, and genes that are expressed will allow them to communicate with each other within and between species in a comparable manner [56]. In addition, the QS process also plays a role in regulating the functions of the cells, such as resistance to antibiotics, spore formation, toxin production, and mobility regulation [57]. Thus, QS inhibition (QSI) is a well-known approach to control bacterial infections since the virulence/pathogenicity of bacteria would be reduced as well as biofilm formation can be inhibited. It has been demonstrated in several recent studies that the QS mechanism is intimately related to the development of bacterial resistance [58]. Therefore, inhibiting the QS mechanism has the potential to represent a potentially promising new antibacterial strategy that can both inhibit bacterial resistance development and inhibit the expression of virulence genes associated with the density of a given pathogen population within a given population [59]. The present study also exhibited anti-QS properties of Sumra honey against *C. violaceum* and *P. aeruginosa* since the production of violacein and pyocyanin was inhibited.

Aside from its antimicrobial properties, honey is also thought to have an antioxidant activity that is important for the health of humans [60]. Honey has been shown to exert chemo-preventive effects against cancer through the modulation of oxidative stress, as one of the mechanisms through which it can exert anticancer activity [61]. Three different methodologies, such as DPPH, ABTS^•+^, and β-carotene bleaching assay, were used in this study to assess the ability of Sumra honey to scavenge respective free radicals. According to the results of these tests, the IC_50_ values for each of the three assays are as follows: 5.4 mg/mL for the ABTS^•+^, 7.7 mg/mL for the DPPH, and 20 mg/mL for the β-carotene bleaching test. These values are in accordance with other antioxidant studies [60,62,63]. Compared to the literature, Sumra honey displayed the lowest IC_50_ value. There are several honeys that have been reported to contain antioxidant activity derived from mint, herbs, and acacia trees as botanical sources. Nevertheless, unlike IC_50_ values obtained against DPPH, ABTS^•+^ and β-carotene bleaching assays would suggest different compounds may be responsible for the scavenging of the respective radicals due to the fact that various solvents were used for these assays. A study of the antioxidant activity of Manuka honey has shown that it is one of the less potent honey types when it comes to DPPH assays, but when it comes to ABTS assays, it has proven to be the most potent among other honey types [64]. Based on these findings, it can be concluded that Manuka honey’s antioxidant activity is mainly due to the presence of hydrophilic compounds [64]. Manuka honey has been reported to contain flavonoids, methyl syringate, and polyphenols as the principal antioxidant compounds [65,66]. In other studies, the IC_50_ values of Manuka honey against DPPH and ABTS assays also showed a great deal of variation (from ~5–45 mg/mL), which is more likely due to the area of origin of the honey [67]. Accordingly, current data could mean that Sumra honey derived from the Saudi region can display antioxidant levels that are equivalent or superior to Manuka honey.

## 4. Materials and Methods

### 4.1. Screening of Antibacterial and Antifungal Activity of Honey Sample

#### 4.1.1. Honey Sample, Bacterial, and Fungal Strains

The Sumra honey was purchased from a Saudi Arabian honey company (Alamari Honeys, Al-Baha, Saudi Arabia), which was tested in a regional reference laboratory (Honey Quality Laboratory, Beekeepers Cooperative Society, Albaha, Saudi Arabia) to ensure its authenticity in accordance with Saudi Arabian Standard Organization guidelines, then it was transferred to the laboratory for further evaluation. There was no sign of granulation, fermentation, or contamination in the honey. The honey was stored at a regular laboratory temperature for further study.

A series of clinical wound bacterial isolates were obtained from the King Khalid general hospital in Hail, Saudi Arabia, for in-vitro testing. Several types of isolates were tested, including *Staphylococcus aureus*, *P. aeruginosa*, *E. coli*, and MDR *Acinetobacter baumannii*. Reference bacterial strains were included, such as *B. subtilis* (MTCC 121), *S. aureus* (MTCC 96), *E. coli* (MTCC 9537), and *P. aeruginosa* (MTCC 741) that were obtained from the Microbial Type Culture Collection (MTCC), Chandigarh, India. The obtained bacterial strains were maintained on Muller-Hinton Agar (MHA) plates.

A selection of previously characterized clinically important yeasts isolates as well as laboratory strains were all included. *Candida auris* and *Cryptococcus neoformans* were collected from King Fahad Hospital in Madinah, Saudi Arabia. Other control isolates, such as *Candida krusie* (CCUG 74256) and *Candida albicans* (CCUG 74255) were purchased from the Culture Collection University of Gothenburg (Göteborg, Sweden) by the Special Infectious Agents Unit—BSL3 King Fahd Medical Research Center (KFMRC) at King Abdulaziz University, Jeddah, Saudi Arabia. All pathogenic fungi isolates were stored at -80 in 30% (*v*/*v*) glycerol and Sabouraud dextrose broth.

#### 4.1.2. Determination of Minimum Inhibitory Concentration (MIC) against Bacterial Isolates

A broth dilution method was used to determine the minimum inhibitory concentration (MIC) of Sumra honey against bacterial strains [68]. Using an overnight culture of bacteria in Muller-Hinton broth (MHB), inoculums were prepared from respective bacterial suspension. The honey sample was diluted in sterile distilled water, and aliquots with different concentrations (10–450 mg/mL) were added aseptically into a sterile 96-well microtiter plate (100 μL per well). Cultures of each bacterium (10^8^ CFU/mL) were added to the corresponding wells and incubated for 24 h at 37 °C. After the incubation period, data was recorded based on visual observation for bacterial growth in wells of microtiter plates. The MIC was determined as the lowest concentration that visually had inhibited bacterial growth. Two controls were used; the negative control consisted of the growth medium (MHB) without the presence of any bacteria, while bacterial suspension without any honey sample (untreated) was the positive control. MIC were expressed in mg/mL.

#### 4.1.3. Determination of Minimum Inhibitory Concentration (MIC) against Fungal Isolates

The estimation of MIC was determined using broth microdilution assay to assess anti-fungal activity of Sumra honey. A modified version of the Clinical and Laboratory Standards Institute protocol (NCCLS-M27-A, 2002) was used. Briefly, honey was diluted in sterile water to aseptically prepare different dilations. A 100 µL was aliquoted of all concentrations in a sterile 96 well plat. All wells were then inoculated with 100 µL of fungal suspension prepared in Sabouraud dextrose broth. A negative control (broth only) and a positive control (fungal suspension) were included in each plate. Growth was examined for visual turbidity after 24 h of incubation at 30 °C. The MIC was taken as the lowest concentration of honey that prevented the growth of the tested microorganisms.

#### 4.1.4. Determination of Minimum Bactericidal Concentration (MBC)

The MBC against bacterial strains was performed to determine the bactericidal activity of Sumra honey. Following the MIC assay, the values of MBC were characterized by spreading 5 µL of sample on MHA plates from those wells that showed no signs of growth [68]. Afterwards, the plates were incubated at 37 °C for 18–24 h. At the end of incubation, the MBC was recorded at the lowest concentration, which yielded three or fewer colonies, meaning 99% of the inoculum was killed. The ratio of MBC over MIC was calculated because antibacterial agents are bactericidal if MBC value is not more than four times the MIC value [69].

### 4.2. Antibiofilm Assay of Sumra Honey

To determine the antibiofilm activity of Sumra honey multiple antibiofilm assays were conducted. Inhibition of biofilm was evaluated using a spectroscopic assay as previously described [70]. Cell suspensions (100 µL) of respective bacterial strains (10^8^ CFU/mL) and Sumra honey (1/2 MIC) were added into designated wells of microtiter plates and then incubated at 37 °C for 24 h. After the incubation, planktonic cells were removed by washing wells very delicately with phosphate buffered saline (PBS) (200 µL) (Hi-Media, India). Biofilms developed by adherent cells that were stained with 0.1% crystal violet (100 µL) (Hi-Media, India), followed by incubation at 37 °C for 30 min. PBS was used to wash off the extra stain, and 200 µL of 95% ethanol was added to each stained well, followed by incubation at 37 °C for 15 min to facilitate solubility of used dye. Absorbance was read spectrophotometrically at 590 nm (UV-Visible spectrophotometer, UV-1800, Shimadzu, Japan). The percentage of inhibition was estimated according to the following equation:

[Optical Density (OD) (control) − OD (test)/OD (control)] × 100

### 4.3. Inhibition of Quorum Sensing by Sumra Honey

A well diffusion assay was used to evaluate the anti-quorum sensing activity of Sumra honey against *C. violaceum* (MTCC2656) and *P. aeruginosa* (MTCC2488). Central wells were made into Luria Bertani agar plates using cork-borer and overnight grown culture (100 µL) of both bacterial strains, which was spread over the plates. A total of 60 µL of Sumra honey sample (10 mg/mL) was inoculated into each of the wells, and the plates were incubated at 37 °C for 24 h. On tested bacteria, the inhibition zone was determined after 24 h, which showed anti-QS effects [71]. The MIC value was also determined using the above-mentioned method.

### 4.4. Violacein Inhibition Assay in C. violaceum

This assay was performed to assess the inhibition of violacein by Sumra honey. Violacein pigment produced by *C. violaceum* was extracted and quantified by spectrophotometry during the presence and absence of Sumra honey [72]. A bacterial broth grown for 16–18 h (OD 600 nm = 0.1) was incubated in conical flasks filled with LB broth in the absence and in the presence of Sumra honey (1/2MIC) and incubated at 28 °C for 24 h. The suspensions were centrifuged, and cell pellets were collected and then dissolved in 1 mL Dimethyl sulfoxide (DMSO) for quantification following the incubation process. Bacterial cells were harvested by centrifugation at 10,000 rpm for 10 min. This led to the removal of cell debris, and the absorbance of violacein was measured at 600 nm. A comparison was conducted between the percentage of treated *C. violaceum* and the control sample, using a wavelength of 600 nm to measure difference in the absorbance. The amount of inhibition of violacein production by Sumra honey in the presence of violacein was determined according to the following equation:

% Violacein inhibition = (OD_600_ of control − OD_600_ of treated/OD_600_ of control) × 100

### 4.5. Pyocyanin Inhibition Assay in P. aeruginosa

The pyocyanin inhibition assay was performed to assess the inhibition of pyocyanin by Sumra honey. A culture supernatant of *P. aeruginosa* treated with and without Sumra honey was used to extract pyocyanin according to described methods by Essar et al., (1990). Briefly, 5 mL of treated (1/2MIC) and untreated supernatant of *P. aeruginosa* was first extracted with 3 mL of chloroform and then re-extracted with 1 mL of 0.2 M HCl. This was followed by the transfer of the solution to a glass cuvette for the measurement of absorbance at 520 nm using the above-mentioned method.

### 4.6. Antioxidant Assays of Sumra Honey

The antioxidant activity of Sumra honey was carried out via three different methods: the 1,1-diphenyl-2-picrylhydrazyl (DPPH) radical scavenging activity, 2,2′-azino-bis(3-ethylbenzothiazoline-6-sulfonic acid) (ABTS) radical scavenging activity, and *β*-carotene bleaching assay as described below.

#### 4.6.1. Scavenging Activity of DPPH Free Radicals

Sumra honey was tested for DPPH free radical scavenging activity following the described method [73], with slight modifications. To prepare the DPPH (20 mg/L), 2 mg of DPPH was dissolved in 100 mL of methanol. Concentrations of methanolic honey ranging from 20 to 40 mg/mL were added to 1.5 mL of DPPH solution for testing. Upon incubation at 25 °C for 15 min, the absorbance of the sample was measured at 517 nm. The concentration of ascorbic acid was set as the reference. The scavenging ability of DPPH was determined using the following formula, where A_control_ and A_sample_ are the absorbances of control and sample, respectively. Based on the ascorbic acid calibration curve (0–10 mg/L), the concentration of honey required to scavenge 50% of DPPH∙ The half maximal effective concentration (EC_50_) was determined as follows:

DPPH scavenging activity (%) = (A_control_) − (A_sample_) × 100 (Ac_ontrol_)

A_control_ = absorbance of negative control at the moment of solution preparation.

A_sample_ = absorbance of sample after 6 min.

#### 4.6.2. Scavenging Activity of ABTS Free Radicals

ABTS free radical scavenging activity of Sumra honey was evaluated after minor modifications according to the protocol of Re et al. [74]. Firstly, ABTS solution (7 mM) was prepared in a distilled water (dH_2_O). Then, the ABTS (radical cation) was prepared by mixing the ABTS solution with potassium persulfate (2.45 mM). The generated ABTS was placed at room temperature in the dark for 12–16 h before use. Diluted ABTS was further diluted with dH_2_O until it reached the absorbance of 0.70 at 734 nm. Afterwards, the honey sample (0.07 mL) and ABTS (3 mL) was mixed and incubated for 7 min in the dark and then its absorbance was measured at 734 nm using a spectrophotometer machine. To calculate the antioxidant activity, the following equation was used:

% Inhibition = (A_control_) − (A_sample_) × 100 (A_control_)

where A_c__ontrol_ = absorbance of negative control at the moment of solution preparation and A_sample_ = absorbance of sample after 6 min.

### 4.7. β-Carotene Bleaching Assay

Sumra honey was tested for *β*-carotene bleaching activity according to Ferreira et al. [75] with minor modifications; the *β*-carotene linoleate model system was performed. In a 100 mL round bottom flask, 2 mL of the *β*-carotene (0.2 g/L) in chloroform, 0.02 mL of linoleic acid, and 0.2 mL of Tween 20 was added. The mixture was then infused with 0.2 mL of honey solution. The flask was filled with 50 mL of dH_2_O after the evaporating material had reached dryness under a vacuum at room temperature. To form an emulsion, the mixture was vigorously agitated. Then, 2 mL of the emulsion was transferred to an additional test tube, immediately placed into a water bath at 50 °C and incubated for 16 h. Using a UV-Visible spectrophotometer, the absorbance of the sample was measured every 20 min over a period of 2 h at 470 nm. For the construction of the calibration curve, butylated hydroxytoluene (200 mg/mL) was used as a standard. Results were expressed in an average of three replicates. To calculate the *β*-carotene bleaching activity (CBI), the following formula was used:

CBI (%) = (B_control_) − (B_sample_) × 100 (B_control_)

B_control_ and B_sample_ represent the bleaching rates of *β*-carotene in the control and the sample, respectively.

### 4.8. Gas Chromatography-Mass Spectrophotometry (GC–MS) Analysis

To determine the main components of tested Sumra honey, phytochemicals analyzed by Gas Chromatography-Mass Spectrophotometry (GC–MS) was performed [70]. The Shimadzu Nexis GC-2030 Gas Chromatograph (GC) and QP2020 NX Mass Spectrometer (MS) were used to analyze Sumra honey by GC–MS. For the separation of the analytes SH-Rxi-5Sil (30 m, 0.25 mm ID, 0.25 µm df, Shimadzu) column was used in which temperature was adjusted to 50 °C for 3 min, raised at a frequency of 5 °C per minute up to 250 °C, and finally raised to 270 °C for 10 min. A total of 20 µL of sample was placed in the system, and helium was used as a carrier gas. To determine the probable composition of the honey, the peaks obtained from the GC–MS separation were compared against the National Institute of Standards and Technology (NIST) database to determine the probable composition.

### 4.9. Statistical Analysis

Two-way ANOVA and Bonferroni post hoc tests were conducted to determine any significance (*p* < 0.01) between variables of obtained data. Statistical analysis was performed using GraphPad Prism software (version 5.0).

## 5. Conclusions

This study showed a comprehensive overview of the potential biological activities of Sumra honey that was collected from Saudi Arabia. There was a general bactericidal effect observed against a wide range of all tested clinical and reference bacterial strains, including drug resistant clinical strains of *S. aureus*, *P. aeruginosa*, *E. coli*, and *A. baumannii*. However, antifungal activity was observed mainly toward *C. auris* and *C. albicans*. The inhibition of biofilm formation and the QS system of pathogenic bacteria as well as inhibition of virulence factors, such as violacein and pyocyanin, which are QS-regulated, was also observed in *C. violaceum* and *P. aeruginosa* at ½ MIC concentration of Sumra honey. The antioxidant activity of Sumra honey has also been noted in the fight against different free radicals. Additionally, a GC–MS analysis of Sumra honey revealed that a few different classes of bioactive phytochemical constituents could contribute to the production of the honey’s bioactive properties. As a result of this study, it becomes clear that Sumra honey can be used as a potential product candidate for the production and development of nutraceuticals, functional foods, and even as a potential drug candidate for the treatment and management of various diseases or for therapeutic purposes targeting sensitive bacteria and fungi based on the present findings. However, further investigation is warranted to explore further desired properties of Sumra honey for potential clinical application by conducting in-vivo pharmacological research. In addition, further studies should be conducted in relation to the isolation and characterization of these compounds, as well as the assessment of their biological activities, included in this study to support the above-stated findings from the present study.

## Figures and Tables

**Table 1:** Minimum Inhibitory Concentration (MIC) and Minimum Bactericidal Concentration (MBC) of Sumra honey against clinical and reference bacterial strains.

Bacterial Isolates	Gram-Stain	MIC (mg/mL)	MBC (mg/mL)	MBC/MIC Ratio
**Clinical Isolates**				
*Staphylococcus aureus*	Positive	300	350	1.16
Methicillin-resistant *Staphylococcus aureus- Uni 1*	Positive	300	>450	>1.5
Methicillin-resistant *Staphylococcus aureus- Uni 2*	Positive	250	>450	>1.8
*Pseudomonas aeruginosa*	Negative	250	300	1.2
*Escherichia coli*	Negative	350	>450	>1.28
*Acinetobacter baumannii*	Negative	250	450	1.8
**Reference isolates**				
*Bacillus subtilis* MTCC 121	Positive	80	100	1.25
*Staphylococcus aureus* MTCC 96	Positive	90	150	1.66
*Escherichia coli* MTCC 9537	Negative	100	200	2.0
*Pseudomonas aeruginosa* MTCC 741	Negative	120	250	2.08

**Table 2:** Minimum Inhibitory Concentration (MIC) of Sumra honey against various fungal strains including clinical isolates.

Fungal Strains	MIC (mg/mL)
**Clinical isolates**	
*Candida auris*	600
*Cryptococcus neoformans*	≥1000
**Reference strains**	
*Candida krusei* CCUG 74256	>1000
*Candida albicans* CCUG 74255	700

**Table 3:** Antioxidant of 2,2-diphenyl-1-picryl-hydrazyl-hydrate (DPPH), 2,2′-azino-bis (3-ethylbenzothiazoline-6-sulfonic acid) (ABTS) and β-carotene activities of Sumra honey.

	DPPH IC_50_ (mg/mL)	ABTS IC_50_ (mg/mL)	β-Carotene IC_50_ (mg/mL)
Sumra Honey	7.7	5.4	>20
Ascorbic acid	0.023	0.021	0.017

**Table 4:** Phytochemicals of Sumra honey extract as identified by gas chromatography-mass spectrometry (GC-MS) analysis.

Identified Compound	Class	Area (%)	Retention Time [min]	Molecular Weight [g/mol]
5-Methyl-2-ethylamino-2-thiazoline	Amino acids	10.59	7.223	144.24
4-Hydroxy-1-[4-(hydroxymethyl)-3,6- dioxabicyclo[3.1.0]hexan-2-yl]-5-methylpyrimidin-2-one	Organic compound	3.93	8.515	152.15
2-chloro-Propanoic acid	Organic Acids	3.31	1.555	108.52
Cyclohexanone	Ketone	2.84	11.63	98.14
4-[3-(4-Fluorobenzyloxy)propyl]-1H-imidazole	Fatty acids	2.11	14.124	234.27
Dimethyl (R)-(+)-malate, O-ethoxycarbonyl-	Organic acids	2.05	8.775	162.14
Bicyclo[2.2.1]heptane-1-carboxylic 7,7-dimethyl-	Acid esters	1.36	11.81	557.6
2,4-Dihydroxy-2,5-dimethyl-3(2H)-furan-3-one	Ketone	1.34	5.623	144.12
1-Methylcyclopropanemethanol	Amino acids	1.18	3.199	86.13
Acetamide	Organic Acids	1.18	3.394	399.4
3,5-Methano-2H-cyclopenta[b]furan-2-one	Organic compound	1.15	12.049	340.16
3H-Pyrazol-3-one, 2,4-dihydro-5-methyl-	Organic compound	0.87	5.049	98.1
Azathymine	Ketone	0.78	6.545	127.1
Oxabicyclo[6.1.0]non-6-en-2-one	Organic compound	0.59	7.58	138.16
3,4-Furandimethanol	Organic alcohol	0.54	13.075	128.13
2-Furanmethanol	Organic alcohol	0.45	5.32	155.19
Levoglucosenone	Ketone	0.41	6.853	126.11
Trispiro[4.2.4.2.4.2.]heneicosane	Organic compound	0.41	12.903	288.5
Cirsiumaldehyde	Aldehyde	0.38	16.925	132.16
2,5-Furandione	Organic acids	0.36	10.381	98.06
Ethanethiol	Alcohol	0.34	2.958	62.14
2-Pyridinemethanol	Alcohol	0.3	9.217	109.13
2-Amino-2-methyl-1,3-propanediol	Organic compound	0.27	2.431	105.14
Cirsiumaldehyde	Aldehyde	0.26	14.317	132.16
Hexadecanoic acid	Fatty acid	0.16	14.847	258.41
1,4-Benzodioxan-5-carboxylic	Organic compound	0.09	10.492	234.13

## Data Availability

Data is contained within the article.

## Supplementary Materials

The following are available online at https://www.mdpi.com/article/10.3390/ph15101212/s1, Figure S1: Gas chromatography—mass spectrometry (GC-MS) analysis of Sumra honey. Peaks of major com-pounds were revealed with different percentage of intensity and retention time (RT).
