# Supplementary material for: The Antimicrobial Effects of Saudi Sumra Honey against Drug Resistant Pathogens: Phytochemical Analysis, Antibiofilm, Anti-Quorum Sensing, and Antioxidant Activities
Source: Pharmaceuticals (Basel). 2022 Sep 30;15(10):1212. doi: 10.3390/ph15101212 (PMC9607359; doi:10.3390/ph15101212)
Supplement: Supplementary file 1 [file pharmaceuticals-15-01212-s001.zip › pharmaceuticals-1912809-supplementary.pdf]

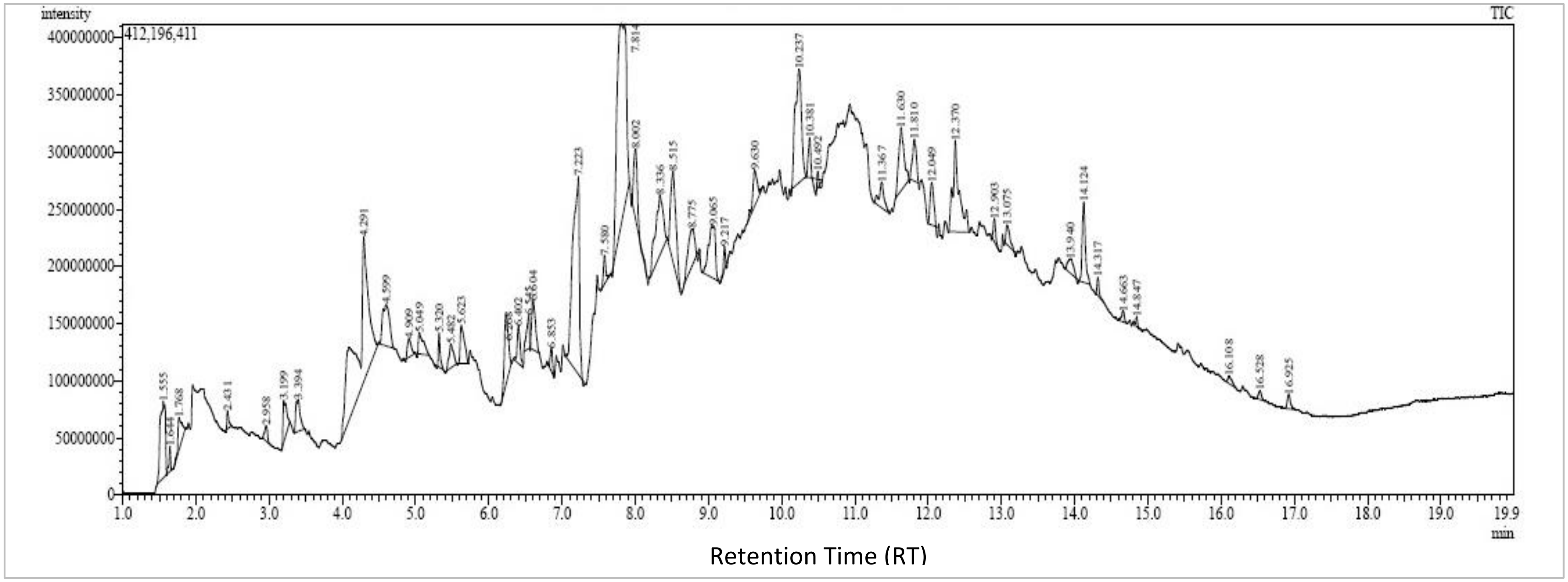

Figure S1. Gas chromatography–mass spectrometry (GC-MS) analysis of Sumra honey. Peaks of major compounds were revealed with different percentage of intensity and retention time (RT).
